# (1*Z*,2*E*)-*N*′-{2-Chloro-1-methyl-2-[2-(4-methyl­phen­yl)hydrazin-1-yl­idene]ethyl­idene}-4-meth­oxy­benzohydrazide

**DOI:** 10.1107/S1600536812008367

**Published:** 2012-03-03

**Authors:** Hatem A. Abdel-Aziz, Hazem A. Ghabbour, Madhukar Hemamalini, Hoong-Kun Fun

**Affiliations:** a Department of Pharmaceutical Chemistry, College of Pharmacy, King Saud University, PO Box 2457, Riyadh 11451, Saudi Arabia; b X-ray Crystallography Unit, School of Physics, Universiti Sains Malaysia, 11800 USM, Penang, Malaysia

## Abstract

The asymmetric unit of the title compound, C_18_H_19_ClN_4_O_2_, contains two mol­ecules, in which the dihedral angles between the benzene rings are 43.60 (12) and 58.65 (13)°. The hydrazine N atoms are twisted slightly out of the planes of the tolyl and meth­oxy­benzene rings: the C—C—N—N and N—N—C—C torsion angles are 171.1 (2) and −174.4 (2)°, respectively, for one mol­ecule and −177.4 (2) and −170.6 (2)°, respectively, for the other. In the crystal, mol­ecules are linked by N—H⋯O and C—H⋯O hydrogen bonds into chains propagating along the *b*-axis direction.

## Related literature

For related structures and background to the bioactivity of hydrazones, see: Chantrapromma *et al.* (2011 ▶); Fun *et al.* (2012 ▶); Abdel-Aziz & Mekawey (2009 ▶); Abdel-Aziz *et al.* (2010 ▶). For reference bond lengths, see: Allen *et al.* (1987 ▶).
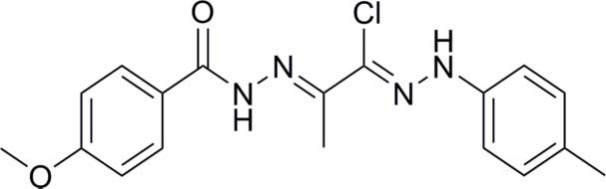


## Experimental

### 

#### Crystal data

C_18_H_19_ClN_4_O_2_
*M* *_r_* = 358.82
Monoclinic, 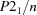

*a* = 10.8081 (6) Å
*b* = 17.6741 (12) Å
*c* = 18.8074 (13) Å
β = 101.292 (5)°
*V* = 3523.1 (4) Å^3^
*Z* = 8
Cu *K*α radiation
μ = 2.08 mm^−1^
*T* = 296 K
0.67 × 0.16 × 0.13 mm

#### Data collection

Bruker SMART APEXII CCD diffractometer
Absorption correction: multi-scan (*SADABS*; Bruker, 2009 ▶) *T*_min_ = 0.338, *T*_max_ = 0.771
23827 measured reflections
6634 independent reflections
4034 reflections with *I* > 2σ(*I*)
*R*_int_ = 0.069

#### Refinement

*R*[*F* ^2^ > 2σ(*F* ^2^)] = 0.050
*wR*(*F* ^2^) = 0.137
*S* = 0.92
6634 reflections
473 parameters
H atoms treated by a mixture of independent and constrained refinement
Δρ_max_ = 0.21 e Å^−3^
Δρ_min_ = −0.27 e Å^−3^

### 

Data collection: *APEX2* (Bruker, 2009 ▶); cell refinement: *SAINT* (Bruker, 2009 ▶); data reduction: *SAINT*; program(s) used to solve structure: *SHELXTL* (Sheldrick, 2008 ▶); program(s) used to refine structure: *SHELXTL*; molecular graphics: *SHELXTL*; software used to prepare material for publication: *SHELXTL* and *PLATON* (Spek, 2009 ▶).

## Supplementary Material

Crystal structure: contains datablock(s) global, I. DOI: 10.1107/S1600536812008367/hb6653sup1.cif

Structure factors: contains datablock(s) I. DOI: 10.1107/S1600536812008367/hb6653Isup2.hkl

Supplementary material file. DOI: 10.1107/S1600536812008367/hb6653Isup3.cml

Additional supplementary materials:  crystallographic information; 3D view; checkCIF report

## Figures and Tables

**Table 1:** Hydrogen-bond geometry (Å, °)

*D*—H⋯*A*	*D*—H	H⋯*A*	*D*⋯*A*	*D*—H⋯*A*
N4*A*—H1N1⋯O1*B*^i^	0.86 (3)	2.47 (3)	3.309 (3)	166 (3)
N1*B*—H1N3⋯O2*B*^ii^	0.84 (3)	2.44 (3)	3.219 (3)	155 (3)
C9*A*—H9*AB*⋯O1*B*^i^	0.96	2.59	3.289 (3)	130
C16*B*—H16*A*⋯O1*A*	0.93	2.49	3.412 (3)	170

Symmetry codes: (i) 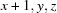; (ii) 
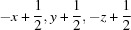
.

**Fig. 1.**
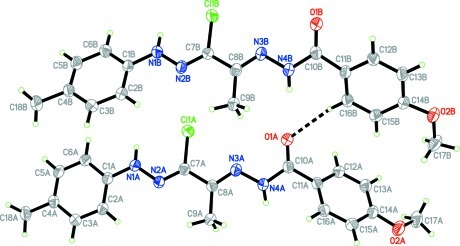
The asymmetric unit of the title compound, showing 30% probability displacement ellipsoids. The C—-H···O hydrogen bond is shown by a dashed line.

**Fig. 2.**
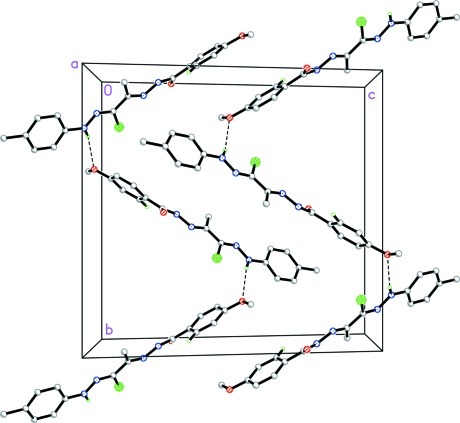
The crystal packing of the title compound (I).

## supplementary crystallographic information

### Comment

As part of our ongoing research on the bioactivity of hydrazones, the title compound (I) was synthesized in order to study and compare its biological activity with other related compounds (Chantrapromma *et al.*, 2011; Fun *et al.*, 2012; Abdel-Aziz & Mekawey, 2009; Abdel-Aziz *et al.*, 2010). Herein we report the synthesis and crystal structure of (I).

The asymmetric unit of the title compound consists of two crystallographically independent molecules, (A & B), as shown in Fig. 1. The bond lengths and angles of molecules A and B agree with each other and are within normal ranges for bond lengths (Allen *et al.*, 1987). The dihedral angles between terminal phenyl rings (C1A–C6A)/(C11A–C16A) and (C1B–C6B)/(C11B–C16B) are 43.60 (12)° and 58.65 (13)° respectively. The hydrazine N atoms are twisted slightly out of the plane of the phenyl and carboxyphenyl rings, C1—N1—N2—C7 and N3—N4—C10—C11 torsion angles are 171.1 (2)° : -174.4 (2)° for molecule A and -177.4 (2)° : -170.6 (2)° for molecule B.

In the crystal, (Fig. 2), the molecules are linked *via* intermolecular N—H···O and C—H···O hydrogen bonds (Table 1), forming supramolecular chains along the *b*-axis.

### Experimental

A mixture of 4-methoxybenzohydrazide (1.66 g, 10 mmol) and 2(*Z*)-2-oxo-*N'*-p-tolylpropanehydrazonoyl chloride (2.11 g, 10 mmol) in absolute ethanol (50 ml) was refluxed for 6 h. The concoction was then left to cool at room temperature. The formed solid was filtered off, washed with ethanol and recrystallized twice from EtOH to afford colourless blocks of the title compound.

### Refinement

Atoms H1N1 and H1N2 were located from a difference Fourier map and refined freely [N—H = 0.83 (3)–0.86 (3) Å]. The remaining H atoms were positioned geometrically [C—H = 0.93 or 0.96 Å] and were refined using a riding model, with *U*_iso_(H) = 1.2 or 1.5*U*_eq_(C). A rotating group model was applied to the methyl groups

### Crystal data

**Table:**

C_18_H_19_ClN_4_O_2_	*F*(000) = 1504
*M**_r_* = 358.82	*D*_x_ = 1.353 Mg m^−^^3^
Monoclinic, *P*2_1_/*n*	Cu *K*α radiation, λ = 1.54178 Å
Hall symbol: -P 2yn	Cell parameters from 682 reflections
*a* = 10.8081 (6) Å	θ = 3.5–50.1°
*b* = 17.6741 (12) Å	µ = 2.08 mm^−^^1^
*c* = 18.8074 (13) Å	*T* = 296 K
β = 101.292 (5)°	Block, colourless
*V* = 3523.1 (4) Å^3^	0.67 × 0.16 × 0.13 mm
*Z* = 8	

### Data collection

**Table:**

Bruker SMART APEXII CCD diffractometer	6634 independent reflections
Radiation source: fine-focus sealed tube	4034 reflections with *I* > 2σ(*I*)
Graphite monochromator	*R*_int_ = 0.069
φ and ω scans	θ_max_ = 70.8°, θ_min_ = 3.5°
Absorption correction: multi-scan (*SADABS*; Bruker, 2009)	*h* = −11→12
*T*_min_ = 0.338, *T*_max_ = 0.771	*k* = −20→21
23827 measured reflections	*l* = −22→22

### Refinement

**Table:**

Refinement on *F*^2^	Primary atom site location: structure-invariant direct methods
Least-squares matrix: full	Secondary atom site location: difference Fourier map
*R*[*F*^2^ > 2σ(*F*^2^)] = 0.050	Hydrogen site location: inferred from neighbouring sites
*wR*(*F*^2^) = 0.137	H atoms treated by a mixture of independent and constrained refinement
*S* = 0.92	*w* = 1/[σ^2^(*F*_o_^2^) + (0.0723*P*)^2^] where *P* = (*F*_o_^2^ + 2*F*_c_^2^)/3
6634 reflections	(Δ/σ)_max_ < 0.001
473 parameters	Δρ_max_ = 0.21 e Å^−^^3^
0 restraints	Δρ_min_ = −0.27 e Å^−^^3^

### Special details

**Table:**

Geometry. All s.u.'s (except the s.u. in the dihedral angle between two l.s. planes) are estimated using the full covariance matrix. The cell s.u.'s are taken into account individually in the estimation of s.u.'s in distances, angles and torsion angles; correlations between s.u.'s in cell parameters are only used when they are defined by crystal symmetry. An approximate (isotropic) treatment of cell s.u.'s is used for estimating s.u.'s involving l.s. planes.
Refinement. Refinement of F^2^ against ALL reflections. The weighted R-factor wR and goodness of fit S are based on F^2^, conventional R-factors R are based on F, with F set to zero for negative F^2^. The threshold expression of F^2^ > 2σ(F^2^) is used only for calculating R-factors(gt) etc. and is not relevant to the choice of reflections for refinement. R-factors based on F^2^ are statistically about twice as large as those based on F, and R- factors based on ALL data will be even larger.

### Fractional atomic coordinates and isotropic or equivalent isotropic displacement parameters (Å^2^)

**Table:**

	*x*	*y*	*z*	*U*_iso_*/*U*_eq_	
Cl1A	0.58879 (6)	0.16734 (5)	0.05740 (4)	0.0709 (2)	
O1A	0.59702 (17)	0.04472 (13)	0.26901 (10)	0.0697 (6)	
O2A	0.93683 (19)	−0.13626 (13)	0.52739 (11)	0.0733 (6)	
N1A	0.7147 (2)	0.17038 (14)	−0.06169 (12)	0.0574 (6)	
H1N2	0.651 (2)	0.1952 (16)	−0.0579 (13)	0.055 (8)*	
N2A	0.77016 (17)	0.12348 (13)	−0.00915 (10)	0.0495 (5)	
N3A	0.71923 (18)	0.06453 (12)	0.16041 (11)	0.0505 (5)	
N4A	0.7690 (2)	0.02089 (13)	0.21952 (11)	0.0505 (5)	
H1N1	0.847 (3)	0.0088 (17)	0.2317 (15)	0.073 (9)*	
C1A	0.7724 (2)	0.18923 (15)	−0.12007 (13)	0.0483 (6)	
C2A	0.8838 (2)	0.15662 (15)	−0.12980 (13)	0.0527 (6)	
H2AA	0.9219	0.1188	−0.0987	0.063*	
C3A	0.9385 (2)	0.18116 (16)	−0.18688 (14)	0.0554 (6)	
H3AA	1.0130	0.1587	−0.1938	0.066*	
C4A	0.8849 (2)	0.23833 (16)	−0.23384 (13)	0.0515 (6)	
C5A	0.7702 (2)	0.26801 (16)	−0.22403 (13)	0.0542 (6)	
H5AA	0.7304	0.3050	−0.2556	0.065*	
C6A	0.7146 (2)	0.24337 (16)	−0.16808 (13)	0.0532 (6)	
H6AA	0.6374	0.2635	−0.1627	0.064*	
C7A	0.7219 (2)	0.11569 (15)	0.04729 (13)	0.0479 (6)	
C8A	0.7762 (2)	0.06449 (14)	0.10652 (12)	0.0465 (6)	
C9A	0.8895 (2)	0.01858 (17)	0.09952 (13)	0.0570 (7)	
H9AA	0.8792	−0.0322	0.1154	0.086*	
H9AB	0.9635	0.0405	0.1289	0.086*	
H9AC	0.8987	0.0180	0.0498	0.086*	
C10A	0.7013 (2)	0.01690 (16)	0.27438 (13)	0.0513 (6)	
C11A	0.7643 (2)	−0.02482 (15)	0.34023 (13)	0.0482 (6)	
C12A	0.7512 (2)	0.00365 (16)	0.40728 (14)	0.0564 (7)	
H12A	0.7036	0.0471	0.4095	0.068*	
C13A	0.8081 (2)	−0.03193 (17)	0.47067 (14)	0.0592 (7)	
H13B	0.8001	−0.0116	0.5152	0.071*	
C14A	0.8764 (2)	−0.09732 (16)	0.46832 (14)	0.0548 (6)	
C15A	0.8862 (2)	−0.12742 (16)	0.40139 (15)	0.0587 (7)	
H15B	0.9297	−0.1725	0.3992	0.070*	
C16A	0.8320 (2)	−0.09081 (16)	0.33831 (14)	0.0562 (6)	
H16B	0.8410	−0.1108	0.2939	0.067*	
C17A	0.9474 (3)	−0.1002 (2)	0.59612 (14)	0.0787 (9)	
H17D	1.0058	−0.1277	0.6319	0.118*	
H17E	0.8662	−0.0991	0.6096	0.118*	
H17F	0.9773	−0.0493	0.5931	0.118*	
C18A	0.9478 (3)	0.26841 (18)	−0.29298 (14)	0.0653 (8)	
H18A	1.0217	0.2389	−0.2948	0.098*	
H18B	0.9715	0.3203	−0.2830	0.098*	
H18C	0.8902	0.2652	−0.3388	0.098*	
Cl1B	0.09492 (6)	0.17833 (4)	0.06847 (4)	0.0690 (2)	
O1B	0.08021 (16)	0.00658 (13)	0.25845 (10)	0.0677 (6)	
O2B	0.41947 (16)	−0.15836 (11)	0.52262 (9)	0.0593 (5)	
N1B	0.2306 (2)	0.18981 (14)	−0.04787 (11)	0.0561 (6)	
H1N3	0.175 (3)	0.2186 (17)	−0.0381 (15)	0.063 (9)*	
N2B	0.27462 (18)	0.13522 (12)	−0.00049 (10)	0.0488 (5)	
N3B	0.21195 (19)	0.06202 (12)	0.16206 (11)	0.0522 (5)	
N4B	0.2637 (2)	0.01366 (14)	0.21748 (11)	0.0553 (6)	
H1N4	0.341 (3)	0.0023 (16)	0.2249 (14)	0.062 (9)*	
C1B	0.2828 (2)	0.20065 (16)	−0.11030 (12)	0.0483 (6)	
C2B	0.3676 (2)	0.15036 (16)	−0.13001 (13)	0.0552 (6)	
H2BA	0.3947	0.1085	−0.1012	0.066*	
C3B	0.4121 (2)	0.16293 (17)	−0.19348 (14)	0.0588 (7)	
H3BA	0.4690	0.1288	−0.2067	0.071*	
C4B	0.3744 (2)	0.22483 (17)	−0.23770 (13)	0.0565 (7)	
C5B	0.2889 (2)	0.27408 (16)	−0.21659 (14)	0.0585 (7)	
H5BA	0.2612	0.3158	−0.2454	0.070*	
C6B	0.2436 (2)	0.26265 (16)	−0.15338 (14)	0.0567 (7)	
H6BA	0.1868	0.2968	−0.1400	0.068*	
C7B	0.2228 (2)	0.12438 (15)	0.05462 (12)	0.0468 (6)	
C8B	0.2722 (2)	0.06806 (14)	0.10995 (12)	0.0476 (6)	
C9B	0.3883 (2)	0.02446 (16)	0.10250 (13)	0.0555 (6)	
H9BA	0.3783	−0.0276	0.1149	0.083*	
H9BB	0.4603	0.0454	0.1345	0.083*	
H9BC	0.4004	0.0277	0.0534	0.083*	
C10B	0.1923 (2)	−0.00721 (16)	0.26657 (13)	0.0518 (6)	
C11B	0.2620 (2)	−0.04923 (15)	0.33097 (13)	0.0486 (6)	
C12B	0.1914 (2)	−0.09340 (17)	0.36911 (13)	0.0574 (7)	
H12B	0.1050	−0.0983	0.3519	0.069*	
C13B	0.2459 (2)	−0.13004 (17)	0.43162 (14)	0.0576 (7)	
H13A	0.1969	−0.1599	0.4560	0.069*	
C14B	0.3738 (2)	−0.12254 (15)	0.45834 (13)	0.0492 (6)	
C15B	0.4464 (2)	−0.08009 (17)	0.42068 (14)	0.0581 (7)	
H15A	0.5330	−0.0759	0.4377	0.070*	
C16B	0.3900 (2)	−0.04350 (17)	0.35718 (14)	0.0568 (7)	
H16A	0.4392	−0.0147	0.3320	0.068*	
C17B	0.5505 (3)	−0.1523 (2)	0.55176 (16)	0.0784 (9)	
H17A	0.5692	−0.1773	0.5979	0.118*	
H17B	0.5734	−0.0999	0.5578	0.118*	
H17C	0.5973	−0.1757	0.5193	0.118*	
C18B	0.4217 (3)	0.2380 (2)	−0.30722 (15)	0.0816 (10)	
H18D	0.4558	0.2882	−0.3070	0.122*	
H18E	0.3532	0.2325	−0.3479	0.122*	
H18F	0.4863	0.2017	−0.3109	0.122*	

### Atomic displacement parameters (Å^2^)

**Table:**

	*U*^11^	*U*^22^	*U*^33^	*U*^12^	*U*^13^	*U*^23^
Cl1A	0.0695 (4)	0.0784 (5)	0.0718 (5)	0.0242 (4)	0.0307 (3)	0.0255 (4)
O1A	0.0578 (11)	0.0935 (16)	0.0601 (12)	0.0181 (10)	0.0175 (8)	0.0240 (11)
O2A	0.0848 (13)	0.0767 (15)	0.0551 (12)	0.0137 (11)	0.0054 (9)	0.0151 (11)
N1A	0.0499 (11)	0.0721 (17)	0.0515 (13)	0.0102 (11)	0.0131 (9)	0.0200 (11)
N2A	0.0478 (10)	0.0576 (13)	0.0422 (11)	0.0008 (9)	0.0068 (8)	0.0061 (10)
N3A	0.0555 (11)	0.0506 (13)	0.0446 (12)	0.0016 (10)	0.0079 (9)	0.0083 (10)
N4A	0.0516 (12)	0.0569 (14)	0.0430 (12)	0.0031 (10)	0.0091 (9)	0.0081 (10)
C1A	0.0472 (12)	0.0531 (15)	0.0433 (13)	−0.0045 (11)	0.0056 (9)	0.0046 (11)
C2A	0.0588 (14)	0.0521 (15)	0.0471 (14)	0.0058 (12)	0.0099 (11)	0.0087 (12)
C3A	0.0561 (13)	0.0606 (17)	0.0509 (15)	0.0065 (12)	0.0137 (11)	0.0020 (13)
C4A	0.0555 (13)	0.0576 (16)	0.0407 (14)	−0.0070 (12)	0.0083 (10)	−0.0016 (12)
C5A	0.0548 (13)	0.0588 (17)	0.0455 (14)	−0.0013 (12)	0.0012 (10)	0.0088 (12)
C6A	0.0457 (12)	0.0640 (17)	0.0491 (15)	−0.0002 (12)	0.0069 (10)	0.0071 (13)
C7A	0.0476 (12)	0.0491 (15)	0.0469 (14)	−0.0040 (10)	0.0087 (10)	0.0025 (11)
C8A	0.0450 (11)	0.0503 (15)	0.0435 (14)	−0.0040 (10)	0.0068 (9)	0.0008 (11)
C9A	0.0544 (14)	0.0682 (19)	0.0479 (15)	0.0111 (13)	0.0085 (11)	0.0093 (13)
C10A	0.0528 (14)	0.0556 (16)	0.0465 (15)	−0.0035 (12)	0.0119 (10)	0.0077 (12)
C11A	0.0469 (12)	0.0524 (15)	0.0459 (14)	−0.0033 (11)	0.0109 (10)	0.0038 (12)
C12A	0.0585 (14)	0.0602 (17)	0.0517 (16)	0.0089 (12)	0.0137 (11)	0.0048 (13)
C13A	0.0667 (16)	0.0651 (19)	0.0457 (15)	0.0036 (13)	0.0111 (12)	0.0011 (13)
C14A	0.0553 (13)	0.0592 (17)	0.0475 (15)	−0.0024 (12)	0.0047 (10)	0.0120 (13)
C15A	0.0618 (15)	0.0535 (16)	0.0625 (17)	0.0078 (13)	0.0166 (12)	0.0056 (14)
C16A	0.0628 (15)	0.0578 (17)	0.0502 (15)	0.0012 (13)	0.0168 (11)	0.0026 (13)
C17A	0.0810 (19)	0.098 (3)	0.0509 (17)	−0.0136 (18)	−0.0027 (13)	0.0153 (18)
C18A	0.0745 (17)	0.075 (2)	0.0491 (16)	−0.0029 (15)	0.0174 (13)	0.0100 (14)
Cl1B	0.0718 (4)	0.0765 (5)	0.0656 (4)	0.0235 (4)	0.0301 (3)	0.0240 (4)
O1B	0.0534 (11)	0.0966 (17)	0.0523 (11)	0.0068 (10)	0.0081 (8)	0.0212 (11)
O2B	0.0651 (10)	0.0595 (12)	0.0490 (10)	−0.0014 (9)	0.0000 (8)	0.0135 (9)
N1B	0.0602 (13)	0.0623 (15)	0.0488 (13)	0.0139 (11)	0.0186 (10)	0.0140 (11)
N2B	0.0524 (10)	0.0527 (13)	0.0403 (11)	0.0001 (9)	0.0066 (8)	0.0068 (10)
N3B	0.0573 (11)	0.0568 (14)	0.0418 (11)	0.0023 (10)	0.0078 (9)	0.0102 (10)
N4B	0.0564 (13)	0.0655 (15)	0.0442 (12)	0.0070 (11)	0.0102 (9)	0.0125 (11)
C1B	0.0458 (12)	0.0575 (16)	0.0412 (13)	−0.0009 (11)	0.0073 (9)	0.0064 (12)
C2B	0.0558 (13)	0.0615 (17)	0.0475 (15)	0.0057 (12)	0.0083 (11)	0.0111 (13)
C3B	0.0536 (13)	0.0712 (19)	0.0532 (16)	0.0059 (13)	0.0142 (11)	−0.0004 (14)
C4B	0.0501 (13)	0.075 (2)	0.0443 (14)	−0.0069 (13)	0.0098 (10)	0.0067 (13)
C5B	0.0628 (15)	0.0601 (18)	0.0531 (16)	−0.0020 (13)	0.0127 (12)	0.0158 (13)
C6B	0.0614 (15)	0.0561 (17)	0.0545 (16)	0.0039 (12)	0.0164 (12)	0.0081 (13)
C7B	0.0458 (12)	0.0499 (15)	0.0441 (13)	0.0010 (10)	0.0073 (10)	0.0026 (11)
C8B	0.0512 (12)	0.0474 (14)	0.0419 (13)	−0.0050 (11)	0.0033 (10)	0.0006 (11)
C9B	0.0599 (14)	0.0603 (17)	0.0460 (15)	0.0044 (12)	0.0097 (11)	0.0064 (13)
C10B	0.0573 (15)	0.0569 (16)	0.0420 (14)	−0.0014 (12)	0.0120 (10)	0.0016 (12)
C11B	0.0515 (13)	0.0531 (15)	0.0405 (13)	−0.0023 (11)	0.0074 (10)	0.0058 (11)
C12B	0.0476 (13)	0.0711 (19)	0.0523 (15)	−0.0068 (12)	0.0068 (10)	0.0112 (14)
C13B	0.0561 (14)	0.0641 (18)	0.0525 (16)	−0.0101 (13)	0.0103 (11)	0.0147 (13)
C14B	0.0569 (13)	0.0458 (14)	0.0439 (14)	0.0028 (11)	0.0074 (10)	0.0045 (11)
C15B	0.0459 (12)	0.0696 (19)	0.0566 (16)	−0.0009 (12)	0.0044 (10)	0.0108 (14)
C16B	0.0523 (13)	0.0661 (18)	0.0526 (15)	−0.0048 (12)	0.0118 (11)	0.0141 (13)
C17B	0.0720 (17)	0.082 (2)	0.070 (2)	−0.0016 (16)	−0.0129 (14)	0.0232 (18)
C18B	0.0762 (19)	0.116 (3)	0.0565 (18)	0.0018 (19)	0.0232 (14)	0.0198 (19)

### Geometric parameters (Å, º)

**Table:**

Cl1A—C7A	1.745 (2)	Cl1B—C7B	1.740 (2)
O1A—C10A	1.216 (3)	O1B—C10B	1.216 (3)
O2A—C14A	1.360 (3)	O2B—C14B	1.368 (3)
O2A—C17A	1.426 (3)	O2B—C17B	1.418 (3)
N1A—N2A	1.338 (3)	N1B—N2B	1.336 (3)
N1A—C1A	1.405 (3)	N1B—C1B	1.412 (3)
N1A—H1N2	0.83 (3)	N1B—H1N3	0.83 (3)
N2A—C7A	1.279 (3)	N2B—C7B	1.285 (3)
N3A—C8A	1.285 (3)	N3B—C8B	1.283 (3)
N3A—N4A	1.373 (3)	N3B—N4B	1.379 (3)
N4A—C10A	1.378 (3)	N4B—C10B	1.365 (3)
N4A—H1N1	0.86 (3)	N4B—H1N4	0.84 (3)
C1A—C2A	1.379 (3)	C1B—C2B	1.379 (3)
C1A—C6A	1.379 (3)	C1B—C6B	1.379 (4)
C2A—C3A	1.392 (3)	C2B—C3B	1.390 (3)
C2A—H2AA	0.9300	C2B—H2BA	0.9300
C3A—C4A	1.391 (4)	C3B—C4B	1.386 (4)
C3A—H3AA	0.9300	C3B—H3BA	0.9300
C4A—C5A	1.391 (4)	C4B—C5B	1.383 (4)
C4A—C18A	1.510 (3)	C4B—C18B	1.513 (3)
C5A—C6A	1.381 (3)	C5B—C6B	1.386 (3)
C5A—H5AA	0.9300	C5B—H5BA	0.9300
C6A—H6AA	0.9300	C6B—H6BA	0.9300
C7A—C8A	1.466 (3)	C7B—C8B	1.463 (3)
C8A—C9A	1.496 (3)	C8B—C9B	1.502 (3)
C9A—H9AA	0.9600	C9B—H9BA	0.9600
C9A—H9AB	0.9600	C9B—H9BB	0.9600
C9A—H9AC	0.9600	C9B—H9BC	0.9600
C10A—C11A	1.487 (3)	C10B—C11B	1.493 (3)
C11A—C16A	1.381 (4)	C11B—C16B	1.378 (3)
C11A—C12A	1.391 (3)	C11B—C12B	1.386 (3)
C12A—C13A	1.381 (3)	C12B—C13B	1.370 (3)
C12A—H12A	0.9300	C12B—H12B	0.9300
C13A—C14A	1.377 (4)	C13B—C14B	1.382 (3)
C13A—H13B	0.9300	C13B—H13A	0.9300
C14A—C15A	1.390 (4)	C14B—C15B	1.378 (3)
C15A—C16A	1.378 (4)	C15B—C16B	1.390 (3)
C15A—H15B	0.9300	C15B—H15A	0.9300
C16A—H16B	0.9300	C16B—H16A	0.9300
C17A—H17D	0.9600	C17B—H17A	0.9600
C17A—H17E	0.9600	C17B—H17B	0.9600
C17A—H17F	0.9600	C17B—H17C	0.9600
C18A—H18A	0.9600	C18B—H18D	0.9600
C18A—H18B	0.9600	C18B—H18E	0.9600
C18A—H18C	0.9600	C18B—H18F	0.9600
			
C14A—O2A—C17A	117.0 (2)	C14B—O2B—C17B	117.8 (2)
N2A—N1A—C1A	121.4 (2)	N2B—N1B—C1B	120.3 (2)
N2A—N1A—H1N2	121.4 (18)	N2B—N1B—H1N3	118.0 (19)
C1A—N1A—H1N2	116.4 (18)	C1B—N1B—H1N3	121.5 (19)
C7A—N2A—N1A	118.9 (2)	C7B—N2B—N1B	119.4 (2)
C8A—N3A—N4A	117.8 (2)	C8B—N3B—N4B	115.9 (2)
N3A—N4A—C10A	117.0 (2)	C10B—N4B—N3B	118.6 (2)
N3A—N4A—H1N1	125 (2)	C10B—N4B—H1N4	119.8 (18)
C10A—N4A—H1N1	115.1 (19)	N3B—N4B—H1N4	121.0 (19)
C2A—C1A—C6A	119.9 (2)	C2B—C1B—C6B	119.9 (2)
C2A—C1A—N1A	122.5 (2)	C2B—C1B—N1B	122.1 (2)
C6A—C1A—N1A	117.6 (2)	C6B—C1B—N1B	118.0 (2)
C1A—C2A—C3A	119.0 (2)	C1B—C2B—C3B	119.2 (2)
C1A—C2A—H2AA	120.5	C1B—C2B—H2BA	120.4
C3A—C2A—H2AA	120.5	C3B—C2B—H2BA	120.4
C4A—C3A—C2A	121.9 (2)	C4B—C3B—C2B	122.0 (3)
C4A—C3A—H3AA	119.0	C4B—C3B—H3BA	119.0
C2A—C3A—H3AA	119.0	C2B—C3B—H3BA	119.0
C5A—C4A—C3A	117.4 (2)	C5B—C4B—C3B	117.3 (2)
C5A—C4A—C18A	120.4 (2)	C5B—C4B—C18B	120.6 (3)
C3A—C4A—C18A	122.1 (2)	C3B—C4B—C18B	122.0 (3)
C6A—C5A—C4A	121.0 (2)	C4B—C5B—C6B	121.5 (3)
C6A—C5A—H5AA	119.5	C4B—C5B—H5BA	119.3
C4A—C5A—H5AA	119.5	C6B—C5B—H5BA	119.3
C1A—C6A—C5A	120.6 (2)	C1B—C6B—C5B	120.0 (3)
C1A—C6A—H6AA	119.7	C1B—C6B—H6BA	120.0
C5A—C6A—H6AA	119.7	C5B—C6B—H6BA	120.0
N2A—C7A—C8A	122.0 (2)	N2B—C7B—C8B	121.2 (2)
N2A—C7A—Cl1A	120.9 (2)	N2B—C7B—Cl1B	121.56 (19)
C8A—C7A—Cl1A	117.15 (16)	C8B—C7B—Cl1B	117.15 (17)
N3A—C8A—C7A	114.6 (2)	N3B—C8B—C7B	115.5 (2)
N3A—C8A—C9A	126.6 (2)	N3B—C8B—C9B	125.9 (2)
C7A—C8A—C9A	118.8 (2)	C7B—C8B—C9B	118.6 (2)
C8A—C9A—H9AA	109.5	C8B—C9B—H9BA	109.5
C8A—C9A—H9AB	109.5	C8B—C9B—H9BB	109.5
H9AA—C9A—H9AB	109.5	H9BA—C9B—H9BB	109.5
C8A—C9A—H9AC	109.5	C8B—C9B—H9BC	109.5
H9AA—C9A—H9AC	109.5	H9BA—C9B—H9BC	109.5
H9AB—C9A—H9AC	109.5	H9BB—C9B—H9BC	109.5
O1A—C10A—N4A	122.4 (2)	O1B—C10B—N4B	122.9 (2)
O1A—C10A—C11A	122.4 (2)	O1B—C10B—C11B	122.3 (2)
N4A—C10A—C11A	115.2 (2)	N4B—C10B—C11B	114.9 (2)
C16A—C11A—C12A	118.5 (2)	C16B—C11B—C12B	118.2 (2)
C16A—C11A—C10A	123.8 (2)	C16B—C11B—C10B	124.5 (2)
C12A—C11A—C10A	117.7 (2)	C12B—C11B—C10B	117.3 (2)
C13A—C12A—C11A	120.7 (3)	C13B—C12B—C11B	121.5 (2)
C13A—C12A—H12A	119.6	C13B—C12B—H12B	119.3
C11A—C12A—H12A	119.6	C11B—C12B—H12B	119.3
C14A—C13A—C12A	120.3 (2)	C12B—C13B—C14B	119.9 (2)
C14A—C13A—H13B	119.8	C12B—C13B—H13A	120.1
C12A—C13A—H13B	119.8	C14B—C13B—H13A	120.1
O2A—C14A—C13A	125.0 (3)	O2B—C14B—C15B	124.3 (2)
O2A—C14A—C15A	115.9 (3)	O2B—C14B—C13B	116.0 (2)
C13A—C14A—C15A	119.2 (2)	C15B—C14B—C13B	119.7 (2)
C16A—C15A—C14A	120.3 (3)	C14B—C15B—C16B	119.8 (2)
C16A—C15A—H15B	119.8	C14B—C15B—H15A	120.1
C14A—C15A—H15B	119.8	C16B—C15B—H15A	120.1
C15A—C16A—C11A	120.8 (2)	C11B—C16B—C15B	121.0 (2)
C15A—C16A—H16B	119.6	C11B—C16B—H16A	119.5
C11A—C16A—H16B	119.6	C15B—C16B—H16A	119.5
O2A—C17A—H17D	109.5	O2B—C17B—H17A	109.5
O2A—C17A—H17E	109.5	O2B—C17B—H17B	109.5
H17D—C17A—H17E	109.5	H17A—C17B—H17B	109.5
O2A—C17A—H17F	109.5	O2B—C17B—H17C	109.5
H17D—C17A—H17F	109.5	H17A—C17B—H17C	109.5
H17E—C17A—H17F	109.5	H17B—C17B—H17C	109.5
C4A—C18A—H18A	109.5	C4B—C18B—H18D	109.5
C4A—C18A—H18B	109.5	C4B—C18B—H18E	109.5
H18A—C18A—H18B	109.5	H18D—C18B—H18E	109.5
C4A—C18A—H18C	109.5	C4B—C18B—H18F	109.5
H18A—C18A—H18C	109.5	H18D—C18B—H18F	109.5
H18B—C18A—H18C	109.5	H18E—C18B—H18F	109.5
			
C1A—N1A—N2A—C7A	171.1 (2)	C1B—N1B—N2B—C7B	−177.4 (2)
C8A—N3A—N4A—C10A	−175.0 (2)	C8B—N3B—N4B—C10B	−166.8 (2)
N2A—N1A—C1A—C2A	5.0 (4)	N2B—N1B—C1B—C2B	8.8 (4)
N2A—N1A—C1A—C6A	−174.0 (2)	N2B—N1B—C1B—C6B	−173.2 (2)
C6A—C1A—C2A—C3A	2.3 (4)	C6B—C1B—C2B—C3B	−0.2 (4)
N1A—C1A—C2A—C3A	−176.7 (3)	N1B—C1B—C2B—C3B	177.8 (3)
C1A—C2A—C3A—C4A	0.9 (4)	C1B—C2B—C3B—C4B	0.3 (4)
C2A—C3A—C4A—C5A	−3.1 (4)	C2B—C3B—C4B—C5B	−0.5 (4)
C2A—C3A—C4A—C18A	176.2 (3)	C2B—C3B—C4B—C18B	−179.3 (3)
C3A—C4A—C5A—C6A	2.3 (4)	C3B—C4B—C5B—C6B	0.7 (4)
C18A—C4A—C5A—C6A	−177.0 (3)	C18B—C4B—C5B—C6B	179.5 (3)
C2A—C1A—C6A—C5A	−3.1 (4)	C2B—C1B—C6B—C5B	0.4 (4)
N1A—C1A—C6A—C5A	175.9 (3)	N1B—C1B—C6B—C5B	−177.7 (3)
C4A—C5A—C6A—C1A	0.8 (4)	C4B—C5B—C6B—C1B	−0.6 (4)
N1A—N2A—C7A—C8A	179.1 (2)	N1B—N2B—C7B—C8B	−176.6 (2)
N1A—N2A—C7A—Cl1A	−2.0 (3)	N1B—N2B—C7B—Cl1B	0.9 (3)
N4A—N3A—C8A—C7A	−176.6 (2)	N4B—N3B—C8B—C7B	−174.9 (2)
N4A—N3A—C8A—C9A	3.0 (4)	N4B—N3B—C8B—C9B	3.5 (4)
N2A—C7A—C8A—N3A	178.0 (2)	N2B—C7B—C8B—N3B	−179.5 (2)
Cl1A—C7A—C8A—N3A	−0.9 (3)	Cl1B—C7B—C8B—N3B	2.9 (3)
N2A—C7A—C8A—C9A	−1.7 (4)	N2B—C7B—C8B—C9B	2.0 (4)
Cl1A—C7A—C8A—C9A	179.34 (19)	Cl1B—C7B—C8B—C9B	−175.62 (19)
N3A—N4A—C10A—O1A	6.4 (4)	N3B—N4B—C10B—O1B	10.0 (4)
N3A—N4A—C10A—C11A	−174.4 (2)	N3B—N4B—C10B—C11B	−170.6 (2)
O1A—C10A—C11A—C16A	138.9 (3)	O1B—C10B—C11B—C16B	−155.4 (3)
N4A—C10A—C11A—C16A	−40.3 (4)	N4B—C10B—C11B—C16B	25.3 (4)
O1A—C10A—C11A—C12A	−39.5 (4)	O1B—C10B—C11B—C12B	20.7 (4)
N4A—C10A—C11A—C12A	141.4 (2)	N4B—C10B—C11B—C12B	−158.7 (2)
C16A—C11A—C12A—C13A	2.1 (4)	C16B—C11B—C12B—C13B	0.6 (4)
C10A—C11A—C12A—C13A	−179.5 (2)	C10B—C11B—C12B—C13B	−175.7 (3)
C11A—C12A—C13A—C14A	−1.5 (4)	C11B—C12B—C13B—C14B	0.8 (4)
C17A—O2A—C14A—C13A	−10.8 (4)	C17B—O2B—C14B—C15B	−1.1 (4)
C17A—O2A—C14A—C15A	169.2 (2)	C17B—O2B—C14B—C13B	179.7 (3)
C12A—C13A—C14A—O2A	179.4 (3)	C12B—C13B—C14B—O2B	177.3 (3)
C12A—C13A—C14A—C15A	−0.7 (4)	C12B—C13B—C14B—C15B	−2.0 (4)
O2A—C14A—C15A—C16A	−177.8 (2)	O2B—C14B—C15B—C16B	−177.5 (3)
C13A—C14A—C15A—C16A	2.2 (4)	C13B—C14B—C15B—C16B	1.7 (4)
C14A—C15A—C16A—C11A	−1.6 (4)	C12B—C11B—C16B—C15B	−0.9 (4)
C12A—C11A—C16A—C15A	−0.6 (4)	C10B—C11B—C16B—C15B	175.1 (3)
C10A—C11A—C16A—C15A	−178.9 (2)	C14B—C15B—C16B—C11B	−0.3 (5)

### Hydrogen-bond geometry (Å, º)

**Table:**

*D*—H···*A*	*D*—H	H···*A*	*D*···*A*	*D*—H···*A*
N4*A*—H1*N*1···O1*B*^i^	0.86 (3)	2.47 (3)	3.309 (3)	166 (3)
N1*B*—H1*N*3···O2*B*^ii^	0.84 (3)	2.44 (3)	3.219 (3)	155 (3)
C9*A*—H9*AB*···O1*B*^i^	0.96	2.59	3.289 (3)	130
C16*B*—H16*A*···O1*A*	0.93	2.49	3.412 (3)	170

Symmetry codes: (i) *x*+1, *y*, *z*; (ii) −*x*+1/2, *y*+1/2, −*z*+1/2.
